# Association between executive functions and gross motor skills in overweight/obese and eutrophic preschoolers: cross-sectional study

**DOI:** 10.1186/s12887-022-03553-2

**Published:** 2022-08-23

**Authors:** Amanda Cristina Fernandes, Ângela Alves Viegas, Ana Cristina Rodrigues Lacerda, Juliana Nogueira Pontes Nobre, Rosane Luzia De Souza Morais, Pedro Henrique Scheidt Figueiredo, Henrique Silveira Costa, Ana Cristina Resende Camargos, Fernanda De Oliveira Ferreira, Patrícia Martins de Freitas, Thiago Santos, Fidelis Antônio da Silva Júnior, Mário Bernardo-Filho, Redha Taiar, Alessandro Sartorio, Vanessa Amaral Mendonça

**Affiliations:** 1 Programa de Pós-Graduação em Reabilitação e Desempenho Funcional (PPGReab), Universidade Federal dos Vales do Jequitinhonha e Mucuri (UFVJM), Campus JK – Rodovia MGT - 367 – Km 583, N°. 5000 – Alto da Jacuba / ZIP, Diamantina, Minas Gerais 39100-000 Brazil; 2 Programa de Pós-Graduação Multicêntrico em Ciências Fisiológicas (PPGMCF), Universidade Federal dos Vales do Jequitinhonha e Mucuri (UFVJM), Diamantina, Minas Gerais Brazil; 3 Centro Integrado de Pesquisa e Pós-Graduação em Saúde (CIPq saúde), Universidade Federal dos Vales do Jequitinhonha e Mucuri (UFVJM), Diamantina, Minas Gerais Brazil; 4 Programa de Pós-Graduação Saúde, Sociedade e Ambiente (PPGSaSA), Universidade Federal dos Vales do Jequitinhonha e Mucuri (UFVJM), Diamantina, Minas Gerais Brazil; 5 Programa de Pós-Graduação em Ciências da Reabilitação, Universidade Federal de Minas Gerais (UFMG), Belo Horizonte, Minas Gerais Brazil; 6 Universidade Federal de Juiz de Fora (UFJF), Juiz de Fora, Minas Gerais Brazil; 7 Programa de Pós-Graduação em Psicologia da Saúde (PPGPSI), Instituto Multidisciplinar em Saúde da Universidade Federal da Bahia (UFBA), Vitória da Conquista, Bahia Brazil; 8 Departamento de Ciências Biológicas, Universidade Federal dos Vales do Jequitinhonha e Mucuri (UFVJM), Diamantina, Minas Gerais Brazil; 9 Laboratório de Vibrações Mecânicas e Práticas Integrativas - LAVIMPI, Departamento de Biofísica e Biometria, Instituto de Biologia Roberto Alcântara Gomes and Policlínica Piquet Carneiro, Universidade do Estado do Rio de Janeiro (UERJ), Rio de Janeiro, RJ Brazil; 10 Université de Reims Champagne Ardenne, MATIM, 51100 Reims, France; 11 Istituto Auxologico Italiano, IRCCS, Division of Auxology and Metabolic Diseases & Experimental Laboratory for Auxo-endocrinological Research, Piancavallo-Verbania, Italy

**Keywords:** Childhood obesity, Gross motor, Object control, Executive functions, Verbal fluency, Tower of Hanoi, Cognitive flexibility, Planning, Child development, Cognitive function

## Abstract

**Background:**

Preschool age (3–5 years old) is a crucial period for children to acquire gross motor skills and develop executive functions (EFs). However, the association between the qualitative gross motor skills and EFs remains unknown in preschoolers, especially among overweight and obese children.

**Methods:**

This was a cross-sectional, exploratory, and quantitative study carried out on 49 preschool children, divided into two subgroups according to their body mass index (overweight/obese: 24; eutrophic [normal weight]: 25). The mean age was 4.59 years. More than half of the sample were boys (55%) and most of the mothers had completed high school (67%) and were class C socioeconomic level (63%). Gross motor skills were assessed using the Test of Gross Motor Development-2, while EFs were evaluated using Semantic verbal fluency (SVF), Tower of Hanoi (TH), Day/Night Stroop, and Delayed Gratification tests. Multiple linear regression models adjusted for sex, age, maternal education, socioeconomic status, quality of the home environment, and quality of the school environment using the stepwise method were executed, considering the cognitive tasks as independent variables and gross motor skills as dependent variable.

**Results:**

The overweight/obese preschoolers showed worse locomotor skills than their eutrophic peers and below average gross motor quotient (GMQ). Overweight/obese girls performed worse in OC skills than boys with excess weight. SVF (number of errors) and TH (rule breaks) explained 57.8% of the variance in object control (OC) skills and 40.5% of the variance in GMQ (*p* < .05) in the overweight/obese children. Surprisingly, there was no significant association between any of the EF tasks and gross motor skills in the eutrophic children.

**Conclusion:**

A relationship between EF tasks (number of errors in SVF and rule breaks in TH) and gross motor skills (OC and GMQ) was demonstrated in the overweight/obese preschoolers, indicating that worse cognitive flexibility, working memory, planning, and problem solving are associated with worse gross motor skills in this population when compared to eutrophic children.

## Introduction

Being overweight or obese, which is a global burden of disease risk, is defined as abnormal or excessive fat accumulation [1]. The presence of obesity in childhood exposes children to an increased risk of obesity in adulthood [2]. The preschool phase (3–5 years old) covers the “adiposity rebound”, in which the amount of fat mass is reduced to a minimum physiological value followed by subsequent rapid weight increase [3]. Furthermore, the preschool period is crucial for children to acquire gross motor skills and develop executive functions (EFs) [4, 5]. Excess weight at this stage in life has been shown to lead to earlier adiposity rebound, which is associated with worse performance of gross motor skills and EFs [3, 6–9].

Gross motor skills are simple movements in daily tasks such as walking, running, jumping, throwing a ball, and kicking a ball. These skills provide a basis for the future acquisition of complex motor skills used in performing activities related to physical fitness, health, and sports [10, 11]. Excess weight can hinder activities that involve the displacement of body mass due to a greater load against gravity, implying biomechanical limitations [12], and possible impairments of the musculoskeletal functions of obese children [13]. In addition, overweight/obese children that are unable to successfully engage in physical challenges may also resist participating in physical activities and overall learning opportunities. Therefore, children with excess adiposity may suffer slower increases in motor proficiency compared to healthy weight children [14].

EFs are higher-order cognitive skills and can be interpreted as central self-regulatory skills that orchestrate basic or domain-specific cognitive processes (e.g., language, attention, sensory input, motor output) to perform reasoning, planning, problem solving [15], and behavior according to the objective [16]. There is general agreement among researchers in the area that the core components of EFs are inhibitory control, working memory, and cognitive flexibility [17], but these core EF components are not yet completely differentiated during the preschool period [18, 19]. Although reasoning, planning and problem solving are developed from the core components of EFs and are therefore more complex EFs [17], preschoolers already show some degree of ability in reasoning, planning, and problem solving [20].

Initially, studies did not include cognitive flexibility in the analyses because preschool children were believed to lack the cognitive development necessary to perform this EF [21, 22]. This is because the development of cognitive flexibility is thought to be dependent on inhibition and working memory, which are still developing in the preschool period [23]. However, several studies have tested structural models of latent variables, including cognitive flexibility. Some studies [24, 25] found that a single latent EF factor provided a good fit to the data for preschoolers, although single-factor models did not outperform all other tested models and were favored for reasons of parsimony. Furthermore, two-factor models present a better fit than models with one or three factors in preschoolers [26]. One such model includes a working memory factor combined with an inhibition-flexibility factor [27–29], while another model includes an inhibition factor combined with a working memory-flexibility factor [26, 30–32]. Thus, all three EFs are present in preschool children, but it is important to consider the rudimentary aspect of EFs in preschoolers [17, 33], especially cognitive flexibility.

Regarding body weight, a previous study found that a greater linear increase in EFs in the preschool phase corresponds to a greater linear decline in body mass index (BMI) [34]. The hypothesis is that there is higher brain energy expenditure at this age, given the increased volume of cortical and subcortical structures. The higher energy requirement for brain development restricts the energy available for body growth, including fat deposition [35]. Thus, if other influences on BMI remain equal, the child with lower peak brain energy demand in the preschool period, or for whom brain energy demand peaks earlier or is of shorter duration, may experience an earlier adiposity rebound. This could lead to a higher lifelong obesity risk [34, 35]. In addition, children with higher general cognition (verbal skills and EFs) at age 4 had a lower likelihood of maintaining an unhealthy weight status between the ages of 4 years and 6 years, and of worsening weight status over time [9]. However, the cross-sectional association between worse EF performance and excess weight, compared to eutrophic (normal weight) children, has only been observed in children over 6 years of age [36, 37]. Before age 6, results may differ according to the cognitive function assessed, which and how many tasks are used to verify cognitive performance [38], and especially if these tasks were adequately adapted for the preschool phase [38, 39].

Mastery of motor tasks requires cognitive skills [40]. In addition to coactivation of the prefrontal cortex, cerebellum, and basal ganglia during various motor and cognitive tasks, motor and cognitive skills have several underlying processes in common, such as sequencing, monitoring, and planning [41]. Performance of complex motor tasks is extremely variable [42] and requires a higher level of EFs than the performance of simple motor tasks [43]. This variability determines whether, and to what extent, cognitive control processes are needed for successful task performance [43]. Overweight/obese preschoolers may have greater performance variability on gross motor tasks because of excess weight [44]. Thus, in overweight/obese preschoolers, motor tasks may be more challenging and present a stronger correlation with cognitive abilities than in normal weight preschoolers [45]. However, to the best of our knowledge, the relationship between EFs and gross motor skills has not been explored in overweight/obese preschoolers. Therefore, it is also not known whether EFs can predict gross motor skills in preschoolers with excess weight.

Studies that evaluated the EFs as predictors of motor performance, are scarce, especially in preschoolers [46–48]. Furthermore, these studies did not specifically focus on core components of EFs or on more complex EFs, but used some measures of EFs for assessment of general cognitive function. Thus, it may be possible to infer the prediction of EFs in preschoolers. The results of a study carried out by the research group of the present study showed that global cognitive function (orientation, attention and working memory, episodic memory, language, and constructional praxis) is an important predictor of gross motor skills in preschoolers, although no specific tests were used to assess the core components of EFs [46]. However, in a sample of children aged 5 to 6 years, Wassenberg et al. [47] found that global cognitive function (general verbal and non-verbal cognitive ability) was not related to motor performance, but rather to separate cognitive measures (visual motor integration, working memory, and number of correct words in the Semantic Verbal Fluency [SVF]), which were predictors of the global measure of motor performance (including fine and gross motor skills). On the other hand, Peyre et al. [48] found that attention and language skills at age 3 contribute to favorable changes in motor skills at age 6. The assessment of language skills also included the number of correct words in the SFV, but alone was not associated with motor skills. The SFV also assesses EFs [49], but the SVF scores needed for more detailed evaluation of EFs (i.e., amount of errors [49]), especially cognitive flexibility, were not evaluated in either study [47, 48]. Regarding planning, although it can also be related to gross motor skills [50], no previous study has exclusively evaluated this relationship in preschoolers. Therefore, a gap remains in relation to the possibility of an association between certain aspects of self-regulation and gross motor skills in the preschool period [43].

Most of the studies that evaluated the relationship between EFs and gross motor skills throughout childhood prioritized understanding the relationship in the opposite direction, that is, gross motor skills as predictors of each of the EFs separately [51–56]. However, in the preschool period, results in eutrophics are controversial [53, 54] as they depend on which EFs were analyzed and how they were evaluated, that is, whether or not cognitive flexibility was included [54–56], and the level of task difficulty [38, 39, 57]. Thus, as the association between EFs and gross motor skills in preschoolers is likely to depend on the level of performance of rudimentary EFs, models that predict EFs separately may find no association or have a smaller effect size [43, 54, 56]. Another critical point is that previous studies often quantitatively evaluated gross motor skills using whole body coordination tasks, involving strength, speed, or agility [43, 55, 58]. However, the qualitative gross motor skills seems more strongly related to EFs than quantitative measures [54]. In addition, studies usually did not control for important sources of stimuli for cognitive [59–61] and gross motor development [62–64] such as the child’s environment (i.e., home and school environment, maternal education and socioeconomic level), nor did they evaluate them for inclusion in statistical analysis as adjustment covariates [46–48, 54, 55].

Thus, for the present study, we considered that the early adiposity rebound is a result of excess adiposity in the critical period for cognitive and motor development. As such, the presence of excess weight may alter the relationship between EFs and gross motor skills. Therefore, the aim of this study was to investigate the association between EFs (including cognitive flexibility and planning) and qualitative gross motor skills in overweight/obese preschoolers. It also aimed to verify whether the possible associations are different in eutrophic preschoolers matched for age, sex, socioeconomic level, and maternal education using adjusted regression models including quality of the home environment and school environment. We hypothesized that executive control processes play a pivotal role for successful motor performance in overweight/obese preschool children, as most motor tasks are challenging in the presence of excess weight, and therefore require more cognitive control [42, 44, 65], reflecting compensatory dependencies between neurocognitive processes [45, 66].

## Methods

### Experimental design and participants

A cross-sectional, exploratory, and quantitative study was carried out with preschoolers aged 3 to 5 years from public schools in the city of Diamantina, MG, Brazil, in the second half of 2019. The university ethics committee granted ethical approval prior to beginning the project, under protocol number 2.355.943. The sample consisted of 49 children divided into two subgroups. The first group was the overweight/obese group, consisting of 24 children with a body mass index (BMI) ≥ 97th percentile (z-score > + 2). The second group included 25 eutrophic children with 3rd ≤ BMI < 85th percentile (− 2 < z-score < + 1) [67], matched for sex, age, socioeconomic level, maternal education, quality of the school environment, and quality of the home environment, which are confounding factors for motor skills and EFs [59–64]. As children from the overweight/obese group were recruited, eutrophic children of the same sex from the same classroom were also recruited so that the groups were composed of children of very similar ages and similar socio-environmental realities (later verified by comparing the socioeconomic level, maternal education, quality of the school environment, and quality of the home environment between the groups – Table 1). The exclusion criteria were children with low birth body weight; premature birth; complications during pregnancy or childbirth or any disease that may impair development; or having had an infectious process in the last 30 days prior to data collection.

**Table 1:** Characteristics of study participants and comparison between groups

Variable	Eutrophic (*n* = 25)	Overweight/Obese (*n* = 24)	φ/φ_c_/r	*p*
Sex, n (%)			0.06	.776 ^a^
Male	13.00 (52.00)	14.00 (58.3)		
Female	12.00 (48.00)	10.00 (41.7)		
Age in *years, median (min-max)*	5.00 (3.00–5.00)	5.00 (3.00–5.00)	0.09	.534 ^b^
Socioeconomic status, n (%)			0.25	.576 ^a^
B	4.00 (16.00)	9.00 (37.50)		
C	18.00 (72.00)	13.00 (54.10)		
D-E	3.00 (12.00)	2.00 (8.30)		
Maternal education, n (%)			0.28	.255 ^a^
Elementary 1	–	2.00 (8.30)		
Elementary 2	3.00 (12.0)	3.00 (12.50)		
High School	18.00 (72.0)	12.00 (50.00)		
Graduated	4.00 (16.0)	7.00 (29.20)		
ECERS in score, *median (min-max)*	2.65 (1.90–2.90)	2.71 (1.90–2.92)	0.03	.809 ^b^
EC_HOME in score, *median (min-max)*	37.00 (30.00–47.00)	41.00 (30.00–50.00)	0.25	.077 ^b^
BMI in kg/m^2^, *median (min-max)*	15.40 (14.10–17.00)	21.60 (19.00–30.10)	0.86	.000 ^b *^
TGMD-2 in score, *median (min-max)*				
Locomotor	9.00 (5.00–12.00)	7.00 (4.00–14.00)	0.39	.007 ^b *^
OC	8.00 (4.00–15.00)	8.00 (6.00–12.00)	0.04	.800 ^b^
GMQ	94.00 (76.00–118.00)	85.00 (73.00–103.00)	0.25	.085 ^b^
SVF in score, *median (min-max)*				
Word production	41.00 (24.00–65.00)	40.5 (26.00–57.00)	0.02	.904 ^b^
Number of errors	1.00 (0.00–4.00)	0.00 (0.00–4.00)	0.14	.321 ^b^
TH, *median (min-max)*				
Number of movements	13.00 (6.00–15.00)	13.00 (5.00–15.00)	0.07	.641 ^b^
Rule breaks	5.00 (2.00–8.00)	5.00 (1.00–8.00)	0.09	.528 ^b^
Delayed Gratification, *median (min-max)*	15.00 (2.00–15.00)	15.00 (1.00–15.00)	0.09	.524 ^b^
Day/Night Stroop, *median (min-max)*	16.00 (3.00–16.00)	14.00 (8.00–16.00)	0.15	.296 ^b^

n: absolute value; %: percentage; Socioeconomic status: high income - A and B, average income - C; low income - D and E; Elementary 1: up to the fifth school year; Elementary 2: up to the ninth school year; High School: three years of intermediate/high school; Graduated: university education; *ECERS* Environment rating scales in early childhood education, *EC_HOME* Early Childhood Home Observation for Measurement of the Environment, *BMI* Body Mass Index, *TGMD-2* Gross Motor Development Test 2, *Locomotor* Standard Score Locomotor, *OC* Standard Score Object Control, *GMQ* Gross Motor Quotient, *SVF* Semantic Verbal Fluency, *TH* Tower of Hanoi

^a^Pearson Chi-Square

^b^Mann Whitney

* Significant difference (*p* < .05)

BMI was calculated based on measurements of body weight and body length, using the World Health Organization (WHO) BMI curves as reference [67, 68]. WHO Anthro software version 3.2.2 (Geneva, Switzerland) was used to calculate BMI for age and sex, expressed in z-scores.

The sample size was estimated using GPower® (Franz Faul, Universitat Kiel, Germany), version 3.1.9.2. F tests were used for the multiple linear regression models. Gross Motor Quotient of The Gross Motor Development Test - second edition (TGMD-2) was considered a dependent variable, and the EFs (inhibition, working memory, and cognitive flexibility) as independent variables, using the correlation values between these variables for preschoolers obtained by Cook et al. [54]. As such, it was possible to calculate the squared multiple correlations (R^2^). The calculated R^2^ value was 0.35, from which we obtained an effect size f^2^ equal to 0.56. Thus, the sample size was estimated at 24 volunteers for each linear regression model, considering a power of 0.80, alpha error set to 5%.

### Measures

#### Socioeconomic status

The Brazilian Economic Classification Criterion was used; this being a questionnaire based on the accumulation of material goods and educational materials. The general socioeconomic classification resulting from this criterion varies from A1 (indicating high economic class) to E (very low economic class) [69].

#### Quality of the home environment

The quality of the home environment where each child lives was assessed through the Home Observation for Measurement of the Environment in Early Childhood (EC_HOME) [70]. The instrument contains 55 items divided into 8 scales: learning materials, language stimulation, physical environment, responsiveness, academic stimulation, modeling, variety, and acceptance. Total score, obtained with the sum of the raw scores of the scales, was used for analysis. The instrument has already been used in a sample of preschoolers in the assessment of psychometric characteristics and demonstrated reliability and validity in a Brazilian sample of preschoolers [71].

#### Quality of the school environment

The quality of the school environment was assessed using the Early Childhood Environmental Rating Scale (ECERS), which consists of 43 items organized into 7 subscales: space and furnishings, personal care routines, language and literacy, learning activities, interactions, program structure, parents and staff. The final score of the scale is given by the average of the raw scores of the seven subscales, the quality interpretation of which is 1-inadequate, 3-minimal (basic), 5-good, and 7-excellent [72, 73]. Studies have shown that the ECERS has good reliability [74, 75]. The instrument has been translated into Portuguese and is widely used in studies with Brazilian preschoolers [73, 75].

#### Gross motor skills

The Test of Gross Motor Development - second version (TGMD-2) was used to evaluate gross motor skills development [76]. TGMD-2 is a standardized norm- and criterion-referenced test for the development of children between 3 and 10 years old, and an instrument with validity and reliability for Brazilian children (indices and values from 0.83 to 0.98) [77]. TGMD-2 is composed of 12 fundamental motor skills, which are subdivided into two subtests: six locomotor skills (run, gallop, hop, leap, horizontal jump, and slide) and six OC skills (striking a stationary ball, stationary dribble (bounce), kick, catch, overhand throw, and underhand roll). The test subject’s score for any skill is assessed as pass/fail (1 or 0) for each of 3 or 4 pattern criteria [76]. The sum of all criteria across all skills within a subtest produces the raw score for each subtest, according to gender and age. Using norm tables, the raw subtest score (Locomotor; OC) is converted to a standard score. Higher scores indicate better quality of movement patterns [76]. The subtest standard scores are combined and converted to an overall Gross Motor Quotient (GMQ) determining a child’s gross motor skills compared to the test’s standardized population. The most reliable score for the TGMD-2 is the GMQ as it is derived from adding the subtest standard scores and converting the sum to a quotient (i.e., a standard score with a mean of 100 and standard deviation of 15) [76]. Children with mean gross motor performance according to TGMD-2 achieve GMQ values between 90 and 110. Therefore, children with GMQ equal to or greater than 90 were considered within the expected range and those who reached up to 89 points were below expected. On the TGMD-2 subtests, children with mean standard scores reach values between 8 and 12. Thus, standardized scores below 8 were considered below expected and equal to or above 8 were within the expected range [76].

#### Assessment of executive functions

EFs were evaluated using the Semantic Verbal Fluency [49], Tower of Hanoi [78], Day/Night Stroop [79], and Delayed Gratification tests [80]. Semantic Verbal Fluency Tests (SVF) have been used to assess vocabulary and speed of mental processing [81, 82], working memory [83], inhibitory control [84], and cognitive flexibility [49]. Participants were asked to name items from five categories (Color, Food, Animals, Toys, and Body Parts) and their answers were orthographically transcribed in real time. The scores for the number of words produced and the number of wrong words in 60 seconds per category were calculated and used in the analyses. SVF has proved to be a valid assessment of both lexical semantic skills and EFs in children [85].

The Tower of Hanoi (TH) is a neuropsychological task used to assess planning and problem solving, in addition to evaluating working memory and inhibitory control [86, 87]. The standard version of the TH consists of three pegs and a pyramid of n-discs, decreasing in size from the bottom to the top. The disks start on one of the pegs, and the objective is to move the entire n-disk pyramid to another peg, subject to two restrictions: only one disk can be moved at a time, and at no point can a larger disk be placed on top of a smaller disk on any peg. Different configurations result in successively more difficult problems, increasing the number of moves needed to reproduce the final goal state configuration; where for each problem n-trials can be given [78]. TH showed high reliability through internal consistency in the study of Humes et al. [88] Based on the hypothesis that preschool children have problem-solving capacity, Klahr & Robinson [78] modified the TH so that it became sensitive to such a capacity. One such modification was the use of a story of monkeys jumping from tree to tree to encourage greater attention and improved grasp of the rules. In the present study, TH was adapted for preschoolers and administered using only two disks [89], with a single trial [90], and one more rule in the story of Klahr & Robinson [78], which was jumping from tree to tree without stopping to jump to the middle tree, making the minimum number of moves equal to 8. For the analysis we used the amount of moves to complete the tower and the number of rule breaks.

The Day/Night Stroop test [79] was used to assess the participants’ ability to inhibit prepotent responses. The Day/Night Stroop test has good internal reliability [91, 92] and good test-retest reliability [93]. The test has a pseudo-random sequence of 16 pictures - eight depicting the sun and the other eight depicting the moon - for which the child was asked to say “night” for the sun image, and “day” for the moon image. The raw score, the number of correct answers (range 0–16), was used in the analysis.

The Delayed Gratification task assesses inhibitory control and self-regulation [80]. Each child’s preference for candy or chocolate as a reward was checked before taking the test, then the children were asked to choose between an immediately available reward of a small amount and a delayed reward of a larger amount, if they waited alone for 15 minutes without ringing the bell. The gratification delay measure was the number of minutes waited [80]. Studies have attested to the construct validity of the task as a measure of delayed gratification in preschoolers [94, 95].

#### Procedures

The first session was carried out at the child’s home with the completion of the survey questionnaires to assess maternal education, socioeconomic data, quality of the home environment (EC-HOME), and anthropometric assessment. The second session was carried out in the school environment, where the daycare (school) environment assessment (ECERS) was applied. In the third session, the parents and the child were referred to the Exercise Physiology Laboratory (LAFIEX), on Campus 2 (UFVJM), to first evaluate the EFs and then the gross motor skills. All children were individually evaluated at the same places. The evaluation of each child lasted around 40 min.

The researchers underwent training to carry out the measurements of weight and height, and to administer the questionnaires, as well as the EF and gross motor tests. To ensure greater reliability, only one examiner for each test battery and session was used, ensuring internal control for the measurement of the outcomes in a sequential study.

### Statistical analysis

Statistical analysis was performed using SPSS 22.0 (Inc., USA). The Shapiro-Wilk test was performed to verify data normality and the Levene test to check for homoscedasticity. Regarding outliers, no variables were detected. Pearson’s chi-squared test was applied to compare the frequency of children in categorical variables (sex, socioeconomic status, and maternal education) between eutrophic and overweight/obese groups. The Mann-Whitney test was used to examine differences between groups. This is because the variables presented non-normal distribution and/or were heteroscedastic. Thus, the means were used only for the classification of gross motor performance according to the TGMD-2 [76]. For the effect size of the comparisons between the groups, φ and φ_c_ (for Pearson’s Chi-squared analyses with categorical or nominal variables, respectively), and r (Mann-Whitney) [96] were calculated. The effect size interpretation was carried out according to Cohen (cut points: small: .10; medium: .30; large: .50) [97]. Bivariate correlations between the variables of interest were explored using Pearson or Spearman correlations according to the normality of the data in each group (eutrophic, overweight/obese). With regard to the confounding variables used in the linear regression models, the results were presented only for age, sex, and maternal education showing a correlation with a cognitive test or the gross motor test. Variables that were significant at a level of *p* < .05 were included in the multiple linear regression analyses as were variables that were decided a priori regardless of significance level (confounding variables). A multiple linear regression was conducted using the forward stepwise method to determine whether EF tasks accounted for significant variance in gross motor skills (locomotor, OC, and GMQ), adjusting for sex, age, maternal education, socioeconomic status, quality of the home environment, and quality of the school environment. Forward stepwise regression was used because it is an appropriate analysis when you have many variables and are interested in identifying a useful subset of the predictors [98]. The F Statistic (probability of F) was used to determine whether a variable should be included in the model. Thus, in each step, the variable that had the smallest *p*-value below the specified limit (*p* < .05) was included in the model. The multiple linear regression assumptions (linearity, absence of multicollinearity, normal distribution, and homoscedasticity of residuals) were met. To assess the relative contribution of cognitive variables to the performance of gross motor skills, the variation partitioning technique was applied [99]. This analysis, performed using R, makes it possible to divide the total percentage of variation explained by shared and individual contributions from the set of predictor variables, in this case, the number of SVF errors and the rule breaks in the TH. The level of significance for analysis was set at 5%.

## Results

The present study involved the participation of 49 children in the age group of 3 to 5 years, divided into two subgroups according to their BMI value (25 eutrophic, 24 overweight/obese). In the present study, the sample was composed mostly of boys; most of the mothers had completed high school and had average socioeconomic level (class C). The sample characteristics and the comparison between groups are reported in Table 1.

The results showed that overweight/obese preschoolers performed significantly lower on locomotor skills than their eutrophic peers with medium effect size, albeit not on object control skills or the overall test (GMQ) (Table 1). Regarding locomotor skills, the overweight/obese preschoolers presented below average scores (mean = 7.63 ± 2.08) according to the norms of the TGMD-2, whereas the eutrophic children presented average scores (mean = 9.08 ± 1.86). For OC skills, both the eutrophic (mean = 8.76 ± 2.69) and the overweight/obese children (mean = 8.33 ± 1.73) presented average performance. In addition, overweight/obese children showed below average gross motor skills (mean = 87.54 ± 9.09), whereas the eutrophic children presented average gross motor skills (mean = 93.20 ± 10.97). Regarding the confounding variables (sex, age, socioeconomic status, maternal education, school environment, and home environment) we did not find any difference between the groups (Table 1).

Bivariate correlations between all variables are presented in Table 2. The correlations varied according to BMI, whereby among the eutrophic preschoolers, age was significantly associated with word production and errors on SVF, and maternal education was significantly associated with movements on TH. Therefore, older children tend to have more word production and a greater number of errors in the SVF, and children who performed fewer movements in the TH tend to be children of mothers with higher education. However, in the presence of excess weight, these correlations were not significant. On the other hand, sex was significantly associated with OC skills only in the overweight/obese group. In this case, girls performed worse (OC skills _median-girls_ = 7.50 ± 2.01; OC skills _median-boys_ = 8.93 ± 1.26; U = 30, z = − 2.38, *r* = 0.49, *p* < .05) and below average (OC skills _mean-girls_ = 7.50 ± 2.01; OC skills _mean-boys_ = 8.93 ± 1.26) according to the norms of the TGDM-2. Overweight/obese girls also had a below average performance in OC skills, unlike eutrophic girls (OC skills _mean_ = 8.58 ± 2.87) and eutrophic boys (OC skills _mean_ = 8.92 ± 2.26), but the median did not show a significant difference between overweight/obese girls and eutrophic girls and boys (eutrophic girls: U = 45.5, z = − 0.98, *r* = 0.21, *p* = .32; eutrophic boys: U = 37, z = − 1.76, *r* = 0.37, *p* = .07).

**Table 2:** Correlation between cognitive tests and gross motor skills in eutrophic and overweight/obesity preschoolers

	1^a^	2^a^	3^a^	4^a^	5^a^	6^a^	7^a^	8^a^	9^a^	10^a^	11^a^	12
1. Age^a^		0.37	**0.49**	−0.24	−0.00	− 0.11	0.31	0.18	0.35	−0.18	−0.19	0.15
2. Sex^a^	0.15		0.17	−0.38	**−0.49**	− 0.28	−0.26	0.05	0.27	0.26	0.16	0.27
3. Maternal education^a^	0.20	0.22		−0.10	0.06	−0.03	0.09	−0.12	0.26	−0.16	0.06	0.03
4. Locomotor	−0.27	−0.25	− 0.28		0.24	**0.79**	−0.20	−0.12	− 0.24	−0.17	− 0.30	−0.26
5. OC	−0.24	−0.16	0.01	0.38		**0.71**	0.18	0.15	−0.22	**−0.67**	**− 0.49**	**−0.46**
6. GMQ	−0.29	−0.20	− 0.12	**0.81**	**0.82**		−0.01	−0.05	− 0.32	**−0.53**	**− 0.48**	**−0.46**
7. Day/Night Stroop^a^	0.22	0.09	−0.27	0.13	−0.14	0.00		0.23	0.06	−0.20	−0.38	− 0.45
8. Delayed gratification^a^	0.39	−0.07	−0.29	− 0.12	−0.35	− 0.31	0.22		0.36	−0.20	−0.12	0.02
9. Word production	**0.71**	0.21	0,20	−0.39	−0.07	− 0.32	0.03	0.34		0.27	0.11	0.06
10. Errors of SVF^a^	**0.41**	0.34	0.08	−0.03	−0.25	−0.20	0.22	0.08	0.17		**0.42**	0.12
11. Movements in TH^a^	0.13	−0.19	**−0.43**	0.06	0.02	0.03	0.14	0.06	−0.18	0.08		**0.43**
12. Rule breaks in TH	0.28	−0.05	− 0.09	−0.00	0.25	0.11	−0.09	0.02	0.13	−0.15	**0.50**	

Bivariate correlations between EF tasks and gross motor skills in eutrophic (lower triangle) and overweight/obese (upper triangle) preschoolers

^**a**^Variables with non-normal distribution for which the Spearman correlation was performed

*OC* Standard Score Object Control, *GMQ* Gross Motor Quotient; *SVF* Semantic Verbal Fluency, *TH* Tower of Hanoi; Values in bold show correlation coefficients with a value of *p* < 0,05

As expected, GMQ was strongly correlated with each component score. Regarding EFs in the overweight/obese group, movements in TH were moderately correlated with errors on SVF. Furthermore, the measurements on EF tasks did not significantly correlate with gross motor skills in the group of eutrophic preschoolers or with locomotor skills in the overweight/obese preschoolers. Nevertheless, a significant moderate correlation was observed in the overweight/obese group between OC skills and the following EF tasks: number of wrong words in the SVF; movements that the child performed to complete TH; and number of rule breaks (TH). With the GMQ variable, significant moderate correlations were observed with the same EF tasks that correlated with OC skills. Although overweight girls had below-average performance in OC skills and a worse mean than overweight/obese boys, and poorer performance in OC skills than normal-weight girls, no correlation between sex and EFs was observed. This indicates that the association between EFs and OC skills may have occurred regardless of gender, which was later confirmed with the results of the multiple linear regression presented below.

In the multiple linear regressions, when the relationship between EFs and locomotor skills was analyzed, no variable remained in the model in either group (Table 3). Regarding OC skills and GMQ, the prediction of EFs was observed only in the overweight/obese group. For both OC skills and GMQ, the first variable inserted in the model was SVF (number of errors) and later TH (rule breaks). Thus, SVF (number of errors) and TH (rule breaks) explained 57.80% of the OC skills variance (*p* < .05) and 40.50% of the GMQ variance (*p* < .05). For each increase in the number of SVF errors, there was a value decrease of 0.59 for OC and 0.47 for GMQ. With respect to TH, for each increase in the number of rule breaks, there was a value decrease of 0.40 for OC and 0.36 for GMQ. Confounding variables of sex, age, maternal education, socioeconomic status, quality of the home environment, and quality of the school environment were included in the stepwise multiple linear regression analyses. These variables were not significant in any model in either group. Therefore, confounding variables were not added to the models (OC skills and GMQ of the overweight/obesity group) and did not affect the β and R^2^ values of the explanatory variables (errors on SVF and rule breaks on TH) (Table 3).

**Table 3:** Multiple linear regression analysis (*forward stepwise*) between cognitive tests and gross motor skills for the overweight/obese group

Predictors	OC			GMQ		
	β	*p*-value	*R*^2^	Β	*p*-value	*R*^2^
			**0.578**			**0.405**
TH (Number of movements)	−0.003	.989		−0.084	.701	
TH (Rule breaks)	−0.400	.011*		−0.364	.044*	
SVF (Number of errors)	−0.597	.000*		−0.477	.010*	
Age in years	−0.007	.964		−0.111	.533	
Sex	−0.133	.402		0.014	.941	
Maternal education	0.048	.747		0.052	.765	
Socioeconomic status	0.151	.302		0.184	.290	
ECERS	0.054	.718		0.075	.671	
EC_HOME	−0.121	.418		0.123	.490	

β: standardized regression coefficient, *OC* Standard Score Object Control, *GMQ* Gross Motor Quotient, *TH* Tower of Hanoi, *SVF* Semantic Verbal Fluency, *ECERS* Environment rating scales in early childhood education, *EC_HOME* Early Childhood Home Observation for Measurement of the Environment; * *p* < .05

The variation partitioning results showed a small overlap between number of SVF errors and number of rule breaks in TH (6.99 and 5.05%, in the explanatory model of OC and GMQ, respectively). The number of SVF errors was the most explanatory independent variable of the variance in OC (35.09%) (Fig. A.1) and GMQ (22.42%) in overweight/obese preschoolers (Fig. B.1).

**Fig. 1:**
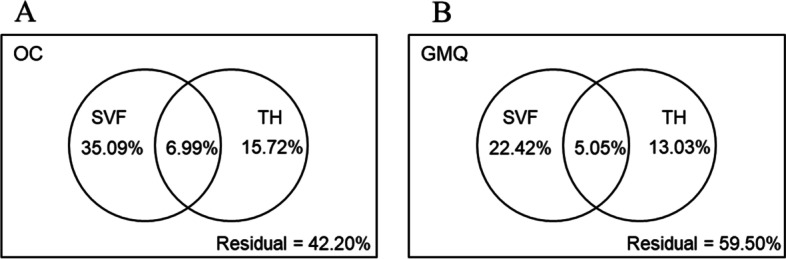
Variance partition analysis. OC: Standard Score Object Control; GMQ: Gross Motor Quotient; SVF: errors of Semantic Verbal Fluency; TH: rule breaks of Tower of Hanoi

## Discussion

This study aimed to examine the association between EFs and gross motor skills in overweight/obese preschoolers and their eutrophic peers controlled for sex, age, maternal education, socioeconomic status, quality of the home environment, and quality of the school environment. Although these associations have been examined in eutrophic preschoolers, to the best of our knowledge, this is the first study to assess the association in overweight/obese individuals in this period of life. In addition, some EFs (cognitive flexibility and planning) in this age group remain poorly explored [38, 50, 54, 55] and most studies have verified the relationship between EFs and quantitatively assessed gross motor skills [43, 55, 58].

### Gross motor skills

A significant difference was observed between the groups for locomotor skills. The worse performance of the overweight/obese children when compared to the eutrophic children can be explained by the biomechanical restrictions: (1) less knee and hip flexion, indicating a more rigid posture during walking; (2) the increase in both the absolute amount of force applied to the joint and the muscular force needed to move the additional mass during ambulation; and (3) the increased compressive and shear forces at the capital femoral growth plate, which can alter the femoral angle in overweight children [100]. These restrictions make locomotor skills more challenging for overweight/obese children than for eutrophics [6]. This result may also be reflected in the below average standardized GMQ score for overweight/obese children, since the GMQ is a composite measure of the Locomotor and OC subscales. The results are in agreement with several studies showing that preschool children with excess weight perform worse in locomotor skills, as expected [101–103].

However, there was no difference in OC skills between eutrophic and overweight/obese preschoolers. OC skills demand high levels of functional coordination and control of objects with the hands, feet, or implements [104] and are important predictors for an active life with greater participation in sports in adolescence and adulthood [11]. It is typically from 6 to 7 years of age that the development of sport-related movement occurs with the motor instruction for OC activities becoming more extensive as children enter primary school [11]. Therefore, in the preschool phase, OC skills are less developed in games that normally require large displacements of the body such as ball games like basketball or soccer [105]. As such, the studies that found worse OC performance in the presence of excess weight were normally evaluating school-age children [106–108]. The execution/learning of OC skills in the preschool period does not require a complete displacement of body weight, but rather the spatial and temporal timing of limb movements [103]. Thus, worse performance in locomotor skills does not necessarily imply worse performance in object control. Although some OC skills on the TGMD-2 include criteria involving some form of body displacement [76], the worse performance in locomotor skills of overweight/obese children in the present study probably did not affect the performance of OC skills. Other studies have also observed the same performance in OC skills among overweight/obese and eutrophic preschoolers [109, 110], although there are also studies that have found worse OC performance in overweight preschoolers [111, 112]. However, these studies did not control for confounding variables.

Regarding gender, overweight/obese girls performed worse in OC skills than boys with excess weight. In addition, only overweight/obese girls performed below average for OC skills. Some studies have observed that girls perform worse in OC skills than boys [46, 113]. This difference can be attributed to different social expectations regarding gender and the nature of the activities offered, or to different innate psychological capacities related to spatial targeting according to gender [114]. However, in the present study, this difference was only observed in the presence of excess weight. Therefore, it is possible that overweight girls feel more inhibited in performing activities involving OC skills than other children [14].

### EFs

Overweight/obese and eutrophic children did not differ in the performance of EF tasks. The impact of excess weight on EFs in the preschool period differs between studies [22, 115, 116]. However, a longitudinal study of eutrophic and obese preschoolers aged 4–6 years found that changes in EF scores associated with BMI occurred only at age 6. However, a higher overall EF score at age 4 was associated with reduced odds of being overweight at age 6 [9]. Therefore, the nature of the relationship between EFs and BMI may be longitudinal rather than cross-sectional in the preschool period [28]. On the other hand, studies show that there is a cross-sectional association between EFs and excess weight in schoolchildren and adolescents [9, 36, 37, 117–120].

### Relationship between EFs and gross motor skills

In the present study, SVF (number of errors) and TH (number of rule breaks) explained 57.80% of the OC skills variance and 40.50% of the GMQ variance in the overweight/obese preschoolers. OC skills require greater cognitive demand than locomotor skills in the preschool period. Locomotor skills emerge first, and young children are therefore more familiar with activities involving locomotion, making EFs less necessary, regardless of BMI [42].

The errors category of the SVF assesses cognitive flexibility and working memory [49]. Thus, the negative association between SVF errors and OC skills in the present study enables the inference that a greater number of SVF errors implies less cognitive flexibility and worse working memory, which are associated with worse performance in OC skills in preschoolers with excess weight. Cognitive flexibility has traditionally been considered a function of the frontal lobe [8], but the prefrontal cortex has reciprocal connections with the cerebellum [121–123]. Thus, studies indicate that the cerebellum, in addition to its role in motor control, is important for language functions, being crucial for verbal fluency, express receptive grammatical processing, and the ability to identify and correct errors in language and writing [124]. In addition, the cerebellum is one of the regions most consistently associated with BMI and obesity [121]. Studies with children over 6 years old show that obese children have larger volumes of white matter in the left cerebellum [125] and decreased white matter organization at the level of the cerebellar peduncles, which is probably due to differences in myelination, axonal density, and/or fiber architecture [8]. These areas have been found to be involved in the frontal-subcortical network connections of the brain responsible for EFs, motor control, and coordination [121, 125]. Therefore, it is possible that excess weight during cognitive and gross motor development contributes to changes in the reciprocal connections between the prefrontal cortex and the cerebellum, favoring a greater interconnection between cognitive flexibility/working memory and OC skills. The relationship with GMQ is likely due to the observed relationship with OC skills. Therefore, less cognitive flexibility and worse working memory are associated with worse performance in OC skills and, consequently, worse GMQ in preschoolers with excess weight.

In eutrophic preschoolers, we found no relationship between SVF and gross motor skills. Two studies investigated the association between SVF word production and motor skills in preschoolers [47, 48], but did not assess BMI and did not assess SVF errors. One of the studies found no association between the variables [48], as observed in both groups in our sample. However, another study [47] found that SVF was a predictor of global motor skills, including gross motor skills. Therefore, the relationship may have been favored by the inclusion of fine motor skills in the total score.

In addition to SVF errors, number of rule breaks in TH also showed a negative association with OC skills and GMQ in the overweight/obese group. Rule breaks in TH lead to wrong moves that are counted in the sum of moves to complete the tower. This explains the observed correlation between rule breaks and movement in TH. Thus, rule breaking results in worse planning and problem solving, increasing the amount of moves needed to complete the tower [43, 126–129]. Therefore, according to the results of the present study, worse efficiency in action planning is associated with worse performance in OC skills and, consequently, worse GMQ in excess weight preschoolers. There are also studies that indicate that action planning is important for successful performance in games involving ball skills [130, 131]. As such, Westendorp et al. [129] carried out a ball skill intervention study with children with learning disorders (age 7–11 years old) and their results showed that ball skills improved with better planning and problem solving. Therefore, in this case, OC skills training was associated with better planning, that is, in the opposite direction to our results. However, it is possible that the relationship between OC skills and planning could be bidirectional and mediated by anticipatory planning. Anticipatory planning is an aspect of motor control necessary for reaching objects that takes into account the future states of the body during the sequence of motor actions while planning the intended maneuver, avoiding uncomfortable postures at the end of object manipulation [132]. A study with normal-weight children aged 5–6 years showed that planning performance in the Tower of London task, unlike inhibitory control and working memory, was a significant predictor of anticipatory planning performance [133]. Thus, if we consider that the quality of OC movement depends on anticipatory motor planning performance [134], it is possible that better planning favors better anticipatory motor planning performance, which in turn enables better performance in OC skills.

On the other hand, similar to that observed in our sample of eutrophic children, a study that evaluated normal-weight children between 3 and 10 years of age found no relationship between cognitive tasks, including TH performance with at least 3 disks (in this case, number of towers completed correctly) and performance on motor tasks that assessed motor planning [135]. In the presence of excess weight, one study found the relationship in school-age children. Obese children solved a lower number of problems, taking less time to plan the movement and more time to perform the movement, and with worse motor skills compared to eutrophic children. Within the total group, better general motor competence was significantly associated with better updating, inhibition control, and planning [136]. Therefore, being overweight can apparently alter the relationship between planning and gross motor skills. This is possible because obese children are normally impulsive [137, 138] and impulsiveness can interfere with planning ability, and, apparently, it may begin in preschool obesity [139].

Impulsivity has long been viewed as a multidimensional construct [140] which includes attentional impulsiveness (tendency to not focus on the task at hand, or thought insertions and racing thoughts), motor impulsiveness (tendency to act on the spur of the moment, or non-perseverance), and non-planning impulsiveness (tendency to not plan and think carefully, or avoid challenging mental tasks) [141]. Prefrontal volumes have been found to be inversely correlated with motor and non-planning impulsivity [142]. In obese children, prefrontal volumes are reduced compared with eutrophic children [143, 144], which, added to cerebellar changes [121, 125], can induce changes in prefrontal-cerebellar connectivity, possibly contributing to the development of impulsivity [145]. Furthermore, planning and problem-solving difficulties, in turn, are affected by other EFs, such as attention, working memory, cognitive flexibility, and inhibitory control, which can also be affected by excess weight [146].

In the present study, we observed a small explanatory percentage of the OC skills variance (6.90%) and the GMQ variance (5.05%) when considering the two tests together (SVF and TH). This was possible because both tests assess some similar EFs, such as working memory and inhibitory control. A study with preschool children observed that better working memory is associated with better performance during strength, speed, and manual dexterity tasks [42]. Other studies demonstrated the impact of obesity on working memory, since obese children performed worse on tasks involving working memory [147, 148]. Although there are also studies that show the association between inhibitory control and motor performance [149], and between inhibitory control and obesity [150], the joint covariance cannot be explained by inhibitory control, at least not alone, since no relationship was found with specific tests for inhibition (Day/Night Stroop and the Delayed Gratification task).

The Day/Night Stroop is considered adequate to be applied in children from 2.5 to 6 years old, although it should be considered that, unlike working memory/cognitive flexibility, the development of inhibitory control has heterotypic continuity [151]. Heterotypic continuity is defined as the “continuity of an inferred genotypic attribute presumed to underlie diverse phenotypic behaviors” [152] or as “the manifestation of the same underlying process through different behavioral presentations at different developmental periods” [153]. In other words, inhibitory control changes during development, from initial dependence on external sources of control to capacity for self-initiated internal forms of control [151]. These changes carry different manifestations of inhibitory control throughout child development and may affect task performance according to age and bio-psycho-social influences [151–153]. In this case, it can be difficult to detect a linear relationship with dependent variables. With respect to the Delayed Gratification task, a recent study demonstrated a new understanding that testing reflects social factors, meaning that children in supportive environments (i.e., warm, affectionate, and open communication in relationships with parents and teachers) can increasingly delay gratification to promote behavioral, social, and academic success throughout their development [154], probably independently of BMI. Thus, it is possible that the inhibitory control assessed by the Delayed Gratification task was impacted by the child’s belief in the expected behavior before the test, which may, consequently, have interfered in the assessment of the relationship between inhibitory control and gross motor skills. Future studies should consider controlling for social support variables, to compare eutrophic and overweight/obese preschoolers, and for evaluating the relationship with gross motor skills.

Finally, no relationship was found between EFs and gross motor skills in eutrophic children, possibly because the performance of gross motor skills in normal-weight children may also be linked to other aspects absent in obese individuals, such as greater body experimentation due to the absence of biomechanical impediments. The addition of these factors may have attenuated the influence of EFs, and, as such, some studies also did not find this relationship in the preschool period [53, 155]. However, some studies did find motor and cognitive skills to be related in eutrophic preschoolers [54, 55]. It is therefore possible that there is not a clear relationship between motor performance and EFs in young children due to the discontinuity in their typical development and the biological coping mechanism of diverting their energy to one specific emerging skill while ignoring others [156]. On the other hand, it has been suggested that stronger associations between developmental domains are expected in children with atypical development, reflecting abnormal dependencies between neurocognitive processes [66]. Nonetheless, in a cross-sectional study, it is not possible to verify if it is obesity that explains the association between EFs and gross motor skills, either because of a smaller linear increase in EFs throughout preschool age, making it difficult for the cognitive performance necessary to perform OC skills, or because obesity affects both cognitive and motor skills independently of each other at preschool age.

#### Strengths and limitations

The present study has several strengths. The analysis of EFs was performed with different and complementary instruments to access more than one aspect of EFs. In addition, we analyzed EFs scarcely explored in preschoolers (cognitive flexibility and planning). For this, we only used instruments adapted for preschool age, including a cognitive test with a lower degree of complexity to assess cognitive flexibility, as well as TH adaptations suggested by the literature that would enable us to verify the planning/problem solving capacity at preschool age. However, more studies are necessary to validate the EF measures for preschool age and to clarify the shared variance between the EFs in these measures to understand the impact of task impurities. In addition, we analyzed the quality of gross motor movements because they are more strongly related to EFs than quantitative measures [54]. Another strength is that the analyses were adjusted for important variables known to impact the development of gross motor skills and EFs. As such, it was possible to detect potential risks that may modify the development of cognitive and motor skills in the presence of excess weight.

Limitations include the absence of normative data from EF tasks for preschoolers. Thus, the evaluation of the EFs did not enable us to verify whether the performance was as expected for the age or not, or whether this could be a determinant for the relationship with gross motor skills [46, 47]. For the TGMD-2, United States norms [76] had to be used for the classification of gross motor performance in Brazil [157, 158], with validated use for Brazilian children [77]. However, cultural differences (i.e., typical United States sports such as baseball) may reflect on the performance of gross motor skills. Thus, it is possible to overestimate or underestimate the performance classification of gross motor skills of Brazilian children, although the associations observed in the present study were not analyzed according to the classification of gross motor performance. In addition, the forward stepwise regression results should be considered with caution, although the method was adequate to verify the most important predictor EFs in an exploratory study. Furthermore, our results limit the generalization of the findings because our sample was mostly composed of males, with mothers that had completed high school and were class C economic level.

#### Future directions

New studies should verify whether the relationship between EFs and gross motor skills in overweight/obese preschoolers is dependent on the degree and duration of obesity, and how this relationship progresses with adiposity rebound. In addition, further studies could also aim to verify if, and to what extent, various cognitive stimuli (i.e., playful activities for cognitive development with and without motor stimulation) collaborate with the development of OC skills in obese children during the preschool phase and if this would bring benefits to the execution of OC skills at later ages. Understanding these processes in the preschool phase can contribute to future measures to prevent childhood obesity and its consequences, such as the elaboration of educational guidelines for preschool. Furthermore, because preschooler development is multifactorial and influenced by both biology and the environment [61–66, 117], longitudinal studies are important to confirm whether these associations remain over time.

## Conclusion

Overweight/obese preschoolers performed worse in locomotor skills than their eutrophic peers paired by sex, age, maternal education, and socioeconomic status, but demonstrated the same performance as their peers in OC and EF skills. We only found a relationship between EF tasks (number of SVF errors and TH rule breaks) and gross motor skills (OC and GMQ) in overweight children, indicating that worse cognitive flexibility, working memory, planning, and problem solving are associated with worse gross motor skills in this population compared to eutrophic children.

## Data Availability

The datasets used and/or analyzed during the current study are available from the corresponding author on reasonable request.
